# Foreign Summary

**Published:** 1839-09-28

**Authors:** 


### FOREIGN SUMMARY.

### Velpeau's Clinical Lectures on Ophthalmia.

No. II.

*Mucous Blepharitis.*

Mucous blepharitis is the "conjunctivitis palpebralis" of authors. When the inflammation does not invade the entire conjunctiva, it shows itself under the form of regular or irregular patches, the colour of which varies between a deep and a pale red. In all cases, the principal symptoms are the following:—The patient at first feels a prickling sensation in the inflamed part. This sensation he compares to that which any extraneous body (such as sand, for instance) would produce, were it placed between the ocular conjunctiva and the internal surface of the eyelids. On everting the palpebræ, you will perceive a great number of small vessels interlaced in every direction. The colour and size of these vessels are not uniformly the same. They appear gradually smaller as they approach the oculo-palpebral sinus; the redness also becomes less apparent as we recede from the free edge of the eyelid. Their mobility, the freedom with which they glide over the subjacent tissues, indicate clearly that they merely creep, as it were, along the surface of the mucous membrane. These three last characters must not be lost sight of; they will prove exceedingly useful towards establishing the diagnosis.

The functions of the eye are necessarily more or less modified by the inflammation. Generally the secretion of mucus is increased, and this, no doubt, is the reason why the Germans have given to this form of inflammation the name of "catarrhal blepharitis." I shall not, for the present, attempt to prove how futile are the grounds for such a denomination; I will merely observe, that I am perfectly at a loss to know why they should endeavour to render specific a symptom which we meet with in the most healthy patients, and which we can produce whenever we think proper. The mucus, generally clear and limpid, sometimes, however, of a gray purulent colour, is secreted in greater or less abundance, and accumulates in the inner canthus of the eye. In some instances the mucous membrane, swollen and thickened, forms a kind of circular fold around the globe of the eye, thus constituting one of the varieties of chemosis.

Instead of increasing, the secretion from the conjunctiva may decrease, or even be entirely suppressed. When this is the case, the mucous surface becomes dry and shining, and we have what several authors have called "dry ophthalmia, or taraxis."

Such is the first and least severe form of blepharitis. Under judicious treatment, it is generally cured in five or six days, and left to itself, disappears in from ten to fifteen, providing, however, the cause be removed.

*Glandular Blepharitis.*

The seat of glandular blepharitis, as I have already stated, is in the Meibomian glands. Let us examine the symptoms, by which we may distinguish it from the other forms of inflammation.

The prickling sensation, already described, is but slightly felt, and that only towards the external part of the eyelid. The sensation itself is modified; it seems to the patient as if small grains of sand were adhering to the margin of that organ. The inner surface of the palpebræ does not present reticulated patches, as in the mucous form, but a kind of vascular band, extending transversely, close to the free edge, the convexity of which is directed towards the eye. The vascular band is peculiar, the vessels being fixed and immoveable, and the redness gradually becoming more and more vivid as we approach the free margin of the eyelids. This appearance is at once accounted for, when we consider that the region presenting it is the principal seat of the inflammation. The most characteristic symptom, however, is the secretion from the Meibomian glands; this secretion, of a viscous nature and variable consistence, soon becomes concrete on exposure to the atmosphere, and collects between the cilia and the glandular edge. During sleep, when the eyelids are closed, the mucus hardens and glues them together in such a manner, as to render it nearly impossible for the patient to open his eyes, unless they have been previously moistened with warm water. If this precaution is neglected, some of the eyelashes are generally brought away by the efforts which are necessary, and this gives rise to small abscesses and ulcerations. The internal edge of the eyelid is nearly always rather swollen and prominent. Depending on the hypertrophy of the Meibomian glands, this slightly tumefied appearance of the inner margin will be detected at the first glance by an experienced observer. There is neither photophobia nor epiphora, that is, when the glandular inflammation is unaccompanied by other forms of ophthalmia; indeed, although of more importance than mucous blepharitis, it is by no means a serious disease. It may exist during a lengthened period, and become even indefinite when not properly treated; this again may serve to distinguish it from the mucous form of inflammation. We must also keep in mind, when treating this affection, that the chronic form is extremely difficult to cure, and that, consequently, every effort should be made to get rid of the malady when still in the acute stage.

Sometimes the free edge of the vascular ribbon, which I have described as occupying the external part of the eyelid, becomes covered by a white concretion, of variable thickness. This constitutes the form I have designated under the name of diphterical blepharitis. The nature of the affection is implied by the name I have given to it. The surface of this pseudo-membranous layer occasionally presents small granulations of a silvery hue. This diphterical appearance of the eyelids is worthy of attention, as it constitutes a most unfavourable complication. We may generally expect, when it occurs, the disease to prove long rebellious to the most judiciously directed treatment.

*Granular Blepharitis.*

Granular blepharitis has never been described by authors as a distinct form of inflammation; the granulations it presents having been merely looked upon by them as one of the consequences of catarrhal or purulent ophthalmia. It may, however, be met with both in the acute and chronic state, and easily recognised, the peculiar characters it presents rendering it nearly impossible to confound it with any other disease, once the attention has been directed to its existence. I cannot, indeed, see why the mucous follicles of the palpebral conjunctiva should not be subject to diseases of the same nature as the mucous follicles of similar membranes, as, for instance, those of the lining membrane of the intestinal canal. That the affection has its origin in the mucous follicles will, I think, be clearly demonstrated by the description of the disease; and I feel convinced that the enumeration of the symptoms will leave but little doubt in your minds respecting the seat of the inflammation.

On examining with care the conjunctiva, we find that it presents a great number of exceedingly small granulations. These granulations, from their arrangement, may be compared to the papillæ of the tongue. At first they are perfectly distinct from one another; they soon, however, increase in number, and form a species of network extending over the entire mucous surface of the eyelid. On this granular net-work we also perceive a number of small vascular ramifications, of a pale red colour, interlacing each other in every direction. After a few days' duration, the vascular appearance sometimes disappears, the granulations alone persisting. When this is the case, we may conclude that the disease is assuming the chronic form. The patient suffers but little. The sensation of extraneous bodies between the eye and palpebræ is modified; it no longer appears to be caused by sand, as in mucous blepharitis, but by particles of finely pulverized dust. If the inflammation is severe, the eyelids are generally more or less swollen; indeed, their volume may increase to such an extent as to conceal entirely the globe of the eye. When they present this appearance, we are often surprised on raising the eyelids to find the eye itself perfectly healthy. The disease may attack the upper or lower eyelid only, or both at the same time. When the upper eyelid is alone inflamed, and the inflammation is slight, it is not very easy to recognise the disease, owing to the difficulty experienced in raising the eyelid in such a manner as to examine it freely. This, however, may be attained by drawing the free margin of the eyelid towards the superciliary ridge, and then communicating to the tarsal cartilage a movement of *bascule*, whilst the patient is directed to look downward. There is scarcely any photophobia, and the secretion from the conjunctiva is not abundant.

This form of blepharitis resists with greater tenacity than any other the action of therapeutical agents; it is, indeed, exceedingly difficult, as we shall shortly see, to effect a cure.

### *Ciliary Blepharitis.*

Ciliary blepharitis, known to surgical writers under the name of psorophthalmia, has been considered by some as a symptom of tinea. Such an opinion, however, is purely hypothetical, experience daily pointing out to us its fallacy. This disease is by no means rare, and must have become familiar to you, as we have continually cases in the wards on which you may study it. Although most authors have given but a short and imperfect description of this affection, it is, I assure you, worthy of all the attention you can bestow. Not only are its consequences often extremely disagreeable, when not well treated from the commencement, but it may also give rise to nearly all the different inflammations of the eye. It is principally at the onset, when the symptoms are generally overlooked, that I should advise you to pay attention to this disease. At a later period, when the inflammation has existed for some time, it becomes extremely difficult to get rid of the complaint; indeed, it will often resist every means you may employ. In the first period there is merely slight itching and redness of the external edge of the eyelids. We have no sensation of an extraneous body, no photophobia or shedding of tears, nor are the natural functions of the eye in the least deranged; such are the forerunners of the malady. No one would suppose that this was the first stage of a disease which proves so rebellious to every species of treatment, and which may lead to most of the more serious affections of the eye. Generally, the persons thus affected pay no attention to these symptoms. Soon, however, the base of the cilia becomes covered with small yellowish scales, which the patient brings away on rubbing his eyes. These scales gradually become larger, more adherent, leaving behind them, when they fall, small ulcerations, on which there forms a thin incrustation. These incrustations are in their turn rubbed off, and are replaced each time by others. The small ulcerations furnish a kind of viscous, dark-coloured secretion, which adheres to the cilia, glueing them together in such a manner that several of the cilia uniting, form, as it were, small pencil brushes, giving to the eyelids a very peculiar appearance. If the disease be not then arrested, the cilia, whose roots are destroyed by the progress of the ulcerations, fall gradually, and the eye is sometimes entirely deprived of the protection they should afford. This state of the eyelids, as you may easily imagine, becomes a permanent cause of keratitis and conjunctivitis. The external edge of the margin of the eyelid is then of a dark red colour, and assumes a rounded, tumefied appearance. These are the symptoms ciliary blepharitis presents, when existing alone, without any complication. It is, however, generally combined with the glandular form of inflammation, and this combination gives rise to symptoms which vary according as the one or the other of the two affections predominates. When the inflammation of the ciliary glands is the more severe, the external free edge of the palpebræ being swollen, becomes slightly inverted, and the cilia are turned inwards, against the globe of the eye, thus constituting an entropion, which can only be cured by an operation. If, on the contrary, the inflammation of the Meibomian glands predominates, the symptoms are reversed. It is then the internal free edge of the palpebræ which is swollen, and consequently more or less everted, the cilia are therefore directed outwards. The whole of the cilia will fall off occasionally, leaving a naked surface, in some instances nearly a quarter of an inch in width. It is not a rare occurrence to meet with old people, whose eyelids have been long inflamed, in this state.

### *Purulent Blepharitis of New-born Children.*

This disease has been improperly confounded by ophthalmologists with purulent ophthalmia, properly so called. They describe three forms of purulent ophthalmia: ophthalmia of new-born children, Egyptian ophthalmia, and gonorrhœal ophthalmia. I cannot, however, admit this division. The Egyptian and gonorrhœal ophthalmia do not so much attack the palpebræ as the eye itself; whereas the purulent ophthalmia of children is mostly confined to these organs; nor is it only the part affected which varies in these diseases; the symptoms are also different, and the prognosis is much less unfavourable in the one than in the other two. I shall, therefore, for the present, confine myself to the description of purulent blepharitis, deferring that of the two other forms of purulent inflammation until treating of the eye itself.

Purulent blepharitis of new-born children has been thus called, from its generally attacking children within the first fortnight after birth. It, however, sometimes occurs several months or several years later. I have met with it, at La Pitié, in a child twelve years old.

The etiology of this affection is exceedingly obscure; and although, as I have already stated, I intend deferring the consideration of specific causes until the termination of these lectures, I shall on this occasion depart from the rule I have laid down. Some authors, looking upon the disease as contagious, attribute it to infection from the mother, whom they suppose to be labouring under gonorrhœa or leucorrhœa at the time of birth. Such an explanation of its production, if applied to every case, in my opinion is perfectly inadmissible. Do we not meet with many cases of purulent ophthalmia, although we cannot possibly entertain any suspicion respecting the health of the mother? Have we not already seen that children may be affected with this disease some years after birth? We should also bear in mind that when the child traverses the vagina, the palpebræ are closed, sometimes folded inwards, as it were, towards the eye, and consequently the mucous surface is protected from external agents.

Vitiation of the atmosphere, the aggregation of many individuals in the same locality, bad living, uncleanliness, &c., have been assigned as the causes of purulent blepharitis. They seem, indeed, well calculated to produce such an affection, but I can hardly admit that alone, they suffice to account, in every case, for its appearance. Do we not occasionally meet with this form of ophthalmia in the higher classes of society, although every law of hygiene has been observed? These considerations induce me to confess that in this, as in many other instances, the principal cause has hitherto eluded our observation.

Another question naturally presents itself to us. Are we to consider purulent blepharitis a contagious malady? The facts of which I am now in possession are not sufficiently numerous to authorize my giving a decided opinion on so important a subject. The disease is seen, it is true, in children who are not supposed to have been in communication with others similarly affected; but can we, in these instances, assert with confidence that no communication of any kind has really taken place? The subject deserves to be treated at length; but a serious examination of the different opinions which have been brought forward, would lead me too far. I shall, therefore, proceed to enumerate the symptoms which the disease presents.

The little patient seems at first to be inconvenienced by the light; the eyes are also evidently the seat of considerable formication, the child continually raising its hands to them. The free edges of the palpebræ are red, rather tumefied, covered by a thin layer of white glutinous matter, which unites and forms a kind of viscid mass in the inner angle of the eye. This is the first stage of the disease, and is the period in which the therapeutical agents that we possess against this complaint should be employed, in order to arrest its progress, which may yet be done. The practioner must not, however, be deceived by the mildness of these premonitory symptoms. The inflammation soon attacks the palpebral conjunctiva, the secretion becoming, at the same time, more abundant. This secretion, at first liquid, transparent, and called by ophthalmologists *hydorrhœa*, becomes of greater consistence in the course of a few days, and is then termed *phlegmatorrhœa.* At a later period it assumes a greenish hue, and, from its resemblance to pus, is called *pyorrhœa.* The palpebral conjunctiva now becomes considerably thickened. The eyelids are also tumefied, the upper eyelid covering the lower one, in such a manner as to render it nearly impossible to raise it. The tumefaction is sometimes very considerable, so much so that the eyelids present the appearance of a large abscess. If you could see the inner surface, you would find it covered with granulations, which give it a fungus-like appearance. The purulent secretion, which flows copiously from the eyes, often produces considerable excoriation in the neighbouring parts. In the midst of these severe symptoms we are surprised to find that the eye is generally free from inflammation, and the cornea transparent. Indeed, it has been remarked by many surgical writers, that when the eye itself becomes inflamed, when there is purulent disorganization of the cornea, the patients are able to open their eyes; and that when, on the contrary, the eye remains healthy, the palpebræ are firmly applied to one another. Several attempts have been made to explain this circumstance. Might we not account for it by supposing that, in the first case, the cornea not allowing the rays of light to reach the retina, that membrane is no longer acted upon; whereas, when, on the other hand, the cornea remains transparent, the retina, from its increased sensibility, not being able to bear the impression of light, re-acts on the muscles, which contract, and keep the eyelids in close apposition.

We have now examined the principal symptoms of the different forms of blepharitis. You must not however suppose, from my having described separately each of these forms, that they are always found isolated, perfectly distinct one from another. At the commencement, they often exist separately, but at a later period they are generally combined. Indeed, when we consider how limited is the seat of inflammation, we must acknowledge that it can hardly be otherwise. You will often meet with the mucous, the glandular, and the granular forms in the same patient. Ciliary blepharitis, when it has existed some time, is nearly always accompanied with inflammation of the Meibomian glands.

These diseases may also lead to local affections extremely annoying to the patient; they may even become the causes of much more serious forms of ophthalmia. Ciliary blepharitis, as we have already stated, is frequently followed, when of long duration, by the loss of the cilia; and their absence, besides disfiguring the patient, is a permanent cause of inflammation. Glandular blepharitis gives rise to the small tumours we often observe on the free margin of the eyelids—such as the hordeolum and the various kinds of cysts.

Granular blepharitis keeps up a permanent state of chronic inflammation of the cornea, and, when it presents the fungus-like appearance I have described, sometimes leads to different de generations of the eyelids. Even mucous blepharitis, the mildest of these affections, may invade the ocular conjunctiva, and give rise to conjunctivitis, properly so called.—*London Medical Gazette.*

*Case of Coagulation of the Blood in the Vessels, with Remarks.* By M. ANDRAL.—A woman, 59 years of age, and mother of six children, was admitted into the Hôpital de la Charité, under the care of M. *Andral*, in December last, with symptoms which were supposed to indicate *cancer du pylore et du foie.* She suffered from sharp pains in the epigastrium, especially after taking food, from frequent vomiting, and all the usual distress of a very aggravated dyspepsia. These complaints were of three or four months' duration. Within the last two months, a firm, irregular, and granulated tumour might be discovered in the epigastric and right hypochondriac regions. It was observed that the basilar vein on the left arm formed a hard resisting cord. When pressed upon by the finger, a person could distinctly feel that there were several coagula in it, separable the one from the other, and moveable about within its cavity. The pulse was rapid and small.

The patient was ordered to be bled to a small amount from the left arm. An incision was made in the basilar vein; and a solid, whitish, perfectly-organized coagulum was drawn out from the wound in the vessel. None of the other veins of the arms exhibited any unusual appearance.

The patient became dropsical soon after her admission into the hospital, and died in the last week of the year.

*Dissection.*—The left basilar vein being opened along its whole extent, the coagulum, which occupied its cavity, was found to have no point of adhesion to any part of the vessel's sides. It was firm and so uniformly consistent, that it could be extracted *en un seul cordon* from the vein.

Similar concretions were found in not only the other veins of the left arm, but also in those of both lower limbs, and of the feet. All these vessels, the deep-seated as well as the superficial, were distended with cylindrical fibrinous coagula, of a deep red colour, and which were so dense and firm that they could be readily taken out in one piece. On examining these coagula minutely, they were found to be composed of concentric layers of fibrine, laid one upon the other, the internal being more deeply coloured than the external ones. In the coagulum, which occupied the left femoral vein, the fibrine had at several points become softened, and, as it were, sanious—resembling somewhat softened encephaloid matter: the parietes of the vein, however, at these points did not exhibit any marks of morbid change.

The aorta, as well as the vena cava, contained coagulated blood. The semi-lunar valves at the mouth of the former vessel were beginning to undergo a cartilaginous transformation.

The liver was hypertrophied, and on its convex surface exhibited numerous irregular cancerous masses, separated the one from the other by indurated portions of the hepatic parenchyma. The pylorous too had passed into the state of scirrhous degeneration.

*Remarks.*—The case, which we have now briefly reported, affords most unquestionable evidence that the blood will sometimes undergo a spontaneous coagulation within the vessels during life. What was the cause of this change? It has been usually attributed to the blood being too rich and crassid, from being charged with an excess of fibrine in its constitution. But how can we suppose that such a state should exist in an emaciated patient, who was labouring under an organic disease, and that too of the digestive organs? We are aware indeed that, in some cases, where a patient has been repeatedly bled, the blood has continued to the last to exhibit a decided buffy coat—a state which is commonly believed to indicate an excess in the quantity of its fibrine—but certainly in the last stages of any organic disease it is usually thin and serous. In such a case as the present, there may possibly have been an absorption and admixture of cancerous matter with the circulating fluid, and a consequent morbid affection of the parietes of the vessels. The instances of spontaneous phlebitis coming on independently of all extraneous causes are of occasional occurrence. But the present case cannot well be regarded as of this nature. The patient, it is true, had felt some uneasiness along the course of the basilar vein, and shortly afterwards the vessel was found to be hard and resisting to the finger like a solid cord. It is to be remembered also that none of the veins, in which coagula were discovered, exhibited on dissection any well-marked traces of inflammation of their internal tunic.

M. *Velpeau* and other pathologists have reported several interesting cases of cancerous disease, in which *cette matiere morbide* had been absorbed into, and become mixed with, the torrent of the circulation. It does certainly appear to us to be the most probable explanation of the coagulation of the venous blood in the case above reported, to suppose that the circulating fluid had become extensively altered, and that this alteration was owing, at least in some measure, to the absorption and admixture of the cancerous matter.—*Med. Chir. Rev. from La Lançette Française.*

---

*Cure of a Stubborn Case of Aphonia by means of Ammoniacal Vapours.* By Dr. GERNER.—A young lady was affected, in consequence of a cold, with complete loss of voice, which had already existed three months, notwithstanding all the remedies which were tried. Dr. Gerner, supposing the cause of the affection to be a relaxed state of the mucous membrane of the trachea, at last cured the patient completely in three days by the inhalation of ammoniacal vapours, disengaged from a mixture of a solution of muriate of ammonia and carbonate of potass.—*Brit. and For. Med. Rev., from Zeitschrift für die gesammte Medicin, &c.*

